# Dissolution Study on Grape Polyphenol Hard Gelatin Capsule Dietary Supplements

**DOI:** 10.3389/fnut.2021.780260

**Published:** 2021-11-25

**Authors:** Weiting Lyu, Thamer Omar, Harna Patel, David Rodriguez, Mario G. Ferruzzi, Giulio M. Pasinetti, James W. Murrough, Fernando J. Muzzio, James E. Simon, Qingli Wu

**Affiliations:** ^1^New Use Agriculture and Natural Plant Products Program, Department of Plant Biology and Center for Agriculture and Food Ecosystems, Institute of Food, Nutrition & Health, Rutgers University, New Brunswick, NJ, United States; ^2^Department of Medicinal Chemistry, Ernest Mario School of Pharmacy, Rutgers University, Piscataway, NJ, United States; ^3^Center for Structured Organic Particulate Systems, Chemical and Biochemical Engineering, Rutgers University, Piscataway, NJ, United States; ^4^Eagle Nutritionals, Carlstadt, NJ, United States; ^5^Department of Food, Bioprocessing and Nutrition Sciences, Plants for Human Health Institute, North Carolina State University, Kannapolis, NC, United States; ^6^Department of Neurology, Mount Sinai School of Medicine, New York, NY, United States; ^7^Geriatric Research, Education and Clinical Center, James J. Peters Veterans Affairs Medical Center, Bronx, NY, United States; ^8^Depression and Anxiety Center for Discovery and Treatment, Department of Psychiatry, Icahn School of Medicine at Mount Sinai, New York, NY, United States

**Keywords:** grape seed extract, resveratrol, UHPLC-QQQ/MS, polyphenol, bioavailability

## Abstract

Methods for a dissolution study by ultra-high performance liquid chromatography/triple quadrupole mass spectrometry (UHPLC-QqQ/MS) analysis of grape polyphenol dietary supplements, namely, grape seed extract (GSE) and resveratrol (RSV) capsules, were developed following the guidance of United States Pharmacopeia (USP) <2040>. Two dissolution media, 0.1 N hydrochloric acid (pH 1.2) and 0.05 M acetate buffer (pH 4.6), were evaluated with dissolution apparatus (USP 1), 100 rpm rotation speed, and 900 ml dissolution medium volume. Dissolution profiling was performed over 120 min. Major phenolic compounds of gallic acid, catechin, epicatechin, and procyanidin B2 were quantitated to obtain the dissolution profile of GSE capsules, and trans-RSV was used for RSV capsules. Results indicated that the released trans-RSV for RSV capsules in both of the dissolution media meets the USP standards, and that for the GSE capsules, all the four marker compounds passed the dissolution test in the HCl medium but did not reach a 75% release within 60 min in the acetate buffer. These promising results suggest that the general USP dissolution protocols are adequate for the successful release of RSV capsules in HCl medium and acetate buffer and GSE capsules (in HCl medium), but may be inadequate for GSE capsules in acetate buffer. These results showed that under a low pH of 1.2 (simulated stomach environment), bioactive compounds were released on time from the GSE capsules and met the USP guidelines; however, under a higher pH of 4.6 (simulated duodenum environment), the same biomarkers failed, suggesting the need to further improve the dissolution of GSE over a wider range of pH environments to enhance bioavailability and efficacy.

## Introduction

Grape (*Vitis vinifera*) is native to southern Europe and Western Asia and is grown all over the world today. It has gained a high level of interest from the public health sector because of its numerous active components (1). Grapes are rich in polyphenols, 60–70% of which are found in grape seeds. These active constituents are linked with a broad spectrum of pharmacological and therapeutic benefits, namely, antioxidant, cardioprotective, hepatoprotective, anticarcinogenic, antidiabetic, antimicrobial, and antiviral (2–9). The major phenolic compounds in grape that contribute to the described health benefits are gallic acid, (+)-catechins, (–)-epicatechin, (–)-epicatechin-3-O-gallate, procyanidin dimers (B1–B5), procyanidin C1, and procyanidin B5-3′-gallate (1).

For active ingredients, the United States Pharmacopeia (USP) has established performance standards to detect their release from dosage forms in capsules and tablets that may occur as a result of formulation design or manufacturing processes in order to ensure safety and bioavailability (10, 11). While several quality control tests of dietary supplements (DSs) in the United States have been described in the USP, mandatory testing has not yet been implemented, and the USP does not have a guideline for resveratrol or grape seed extract, although there is one for grape seeds oligomeric proanthocyanidins. The vast majority of marketed DSs do not have USP-approved testing protocols. Importantly, the release of bioactives can be impacted by the manufactured product (e.g., nature of material and composition of the capsule, as well as the insert materials used in the manufacturing such as capsule, cellulose and/or gelatin, commonly used materials, all meeting FDA requirements but not all exhibiting uniform dissolution and breakdown properties) (12). Although the USP general chapter <20 40> describes a set of standardized test protocols tailored for specific combinations of dosage form and ingredient content category and dissolution protocols for vitamin/mineral DSs, it does not specify a dissolution protocol for testing grape-based DSs (10).

As part of the Consortium for Advancing Research on Botanical and Other Natural Products (https://ods.od.nih.gov/Research/Dietary_Supplement_Research_Centers.aspx) Program, grape-derived DSs and the major polyphenols in these products were investigated in support of an NIH-funded U19 clinical trial to ensure that bioactive compounds would be released at specific concentrations over time, thus bridging the link between chemistry, product formulation, and delivery with acceptable predictable release times to achieve a more robust quality control. In this brief research report, methods for dissolution study with UHPLC-QqQ/MS analysis on grape polyphenol DSs, namely, grape seed extract (GSE) capsules and resveratrol (RSV) capsules, were developed following the guidance of the USP <2040>.

## Materials and Methods

### Reagents and Materials

#### Chemical Reagents

Standard compounds, namely, trans-RSV, (+)-catechin, (-)-epicatechin, and gallic acid were purchased from Sigma-Aldrich Chemicals Co. (St. Louis, MO, United States). HPLC-grade water, acetonitrile (ACN), and formic acid (FA) were obtained from Thermo Fisher Scientific Co. (Fair Lawn, NJ, United States). ACS-grade hydrochloric acid, glacial acetic acid, and sodium acetate were also purchased from Thermo Fisher Scientific Co. (Fair Lawn, NJ, United States).

#### Drug Material Sourcing and Manufacturing

Two kinds of grape-based dietary supplements were investigated. MegaNatural® Grape seed polyphenol extract (GSE) was purchased from Polyphenolics Company (Madera, CA, United States), which was produced from grapes that were grown in California, United States, certified by Halal (IFANCA). The final GSE product was processed by hot water extraction at a ratio of 30–50:1 (dry seed: extract). Synthetic trans-resveratrol (RSV) was purchased from BannerBio Nutraceuticals, Inc. (Nanshan District, Shenzhen, China).

Original GSE and RSV materials were delivered to Eagle Nutritionals (Carlstadt, NJ, United States) to prepare the capsules. Briefly, for GSE capsules, 450 mg GSE powder and 50 mg silica were filled into #0 purple/white hard gelatin capsules. For RSV capsules, 450 mg RSV powder was encapsulated using #0 green capsules.

### Equipment

#### Analysis of Dissolution Samples

The instrument used for chemical analysis was an Agilent 1,290 Infinity II UHPLC (Agilent Technologies, Palo Alto, CA, United States) coupled with a 6,470 (Agilent Technologies, Santa Clara, CA, United States) triple quadrupole mass spectrometer with electrospray ionization (ESI) source. Agilent MassHunter Optimizer (version B.07.00) was used for standard compound-related parameter optimization, and MassHunter Workstation software Data Acquisition (version B.08.00) and Quantitative Analysis (version B.07.01) were used for data processing. The column used for compound separation was a Kinetex^TM^ (Phenomenex Inc., CA, United States) C18 column; the particle size was 2.6 μm, and the size was 100^*^2.1 mm.

#### Dissolution Apparatus

All the dissolution testing reported in this study was performed in a 708-DS, eight-spindle, eight-vessel USP dissolution apparatus type I (basket), with automated online UV-Vis measurement (Agilent Technologies, Palo Alto, CA, United States). This apparatus consists of the following: a vessel, which is made of a glass material; a motor; a metallic drive shaft; and a cylindric basket. The vessel is cylindrical with a hemispherical bottom and has a 1-L capacity. The device used has eight vessels and is partially immersed in a water bath. The water bath keeps the temperature inside the vessel at 37 +/−0.5°C during the test. To prevent evaporation, plastic covers on the top of each vessel are used. A rotating shaft is placed in a position that ensures its axis is not more than 2 mm at any point from the vertical axis of the vessel and rotates smoothly. The basket is connected at the bottom of the rotating shaft. The shaft and basket are made of stainless steel. The basket is positioned so that the distance between the inside bottom of the vessel and the bottom of the basket is kept at 25 mm +/−2 mm during the test (10, 11).

### Dissolution Testing

The GSE and RSV capsules were tested for dissolution based on recommendations by the FDA and USP 39 general chapters <2040> and <711> (10, 11). The dissolution apparatus was USP type I (basket method). The capsules were immersed and agitated in 900 ml of an appropriate medium (0.1 N HCl medium, pH 1.2, or pH 4.6 acetate buffer) at 100 rpm rotation speed, and the temperature was 37 ± 0.5°C. The 0.1-N HCl medium was selected to model the pH of the stomach (1.2) while 0.05 M acetate was used to mimic the pH of the duodenum (4.6). Because trans-RSV is light-sensitive, the instrument was protected from light using tin foil. Twelve samples were tested per case. Basket method was preferred in this study, because it can prevent a capsule from floating. During the test, 1 ml of each solution was withdrawn from the dissolution vessel at 5, 10, 20, 30, 45, 60, 90, and 120 min using a syringe equipped with a cannula. The cannula was then removed from the syringe, and a 0.45-mm polytetrafluoroethylene (PTFE) filter was used to filter each sample.

### LC-MS Method

#### Preparation of Standard Solution

Gallic acid, catechin, procyanidin B2, and epicatechin were chosen as markers, because they are important bioactive polyphenols in GSE (1), and based on our previous study, the amount of these four compounds are dominant in GSE. To prepare the reference solution of marker compounds in the GSE capsules (gallic acid, catechin, procyanidin B2, and epicatechin), each standard was accurately weighed and diluted serially using 70% methanol with 0.1% FA to make a 5,000 to ~0.1 ng/ml reference solution. For the RSV capsules, trans-RVS was accurately weighed and diluted serially using 70% methanol with 0.1% FA to make a 5,000 to ~0.1 ng/ml reference solution.

#### Calculation of Marker Compounds in GSE Capsules

To calculate the percentage of marker compounds in each dissolution sample, each of the marker compounds in GSE capsules dissolved in the two-dissolution media (0.1 N HCl medium and pH 4.6 acetate buffer) was quantified. Briefly, one GSE capsule was extracted by sonication for 30 min using each of the dissolution media. The extracted solvent was diluted ten times and then analyzed using the LC-MS method mentioned in Section GSE Capsule Dissolution Samples (LC-MS Method). Three replicates were made in parallel for quality control purposes.

#### GSE Capsule Dissolution Samples (LC-MS Method)

For LC parameters, mobile phase A was 0.1% formic acid (FA) in water, and mobile phase B was 0.1% FA in ACN. The gradient was 2 to 10% B in 0.4 min, and raised to 30% B from 0.4 to 3 min, then raised to 60% B in 0.5 min and dropped to 2% B in 0.1 min, with a flow rate of 0.4 ml/min. The column was equilibrated with 2% B for 0.4 min between injections. The column was thermostatted at 30°C, and the autosampler was set to 4°C. The injection volume was 1 μl.

For MS parameters, nitrogen was used as the nebulizing and drying gas. The nebulizer was set to 35 psi and the drying gas to 300°C with a flow rate of 12 L/min. The sheath gas was set to 250°C with a flow rate of 12 L/min. The nitrogen used for MS electrospray ionization was generated from a Parker Balston NitroFlow60NA nitrogen generator.

Each GSE dissolution sample was diluted 10 times using 70% methanol acidified with 0.1% FA and centrifugated at 12,000 rpm for 10 min. The supernatant was directly injected into UHPLC under dynamic multiple reaction monitoring (dMRM) mode. The dMRM parameters are listed in Table 1.

**Table 1:** Dynamic multiple reaction monitoring (dMRM) parameters of biomarkers in grape seed extract (GSE) and resveratrol (RSV) capsules.

	**Compound**	**Retention time (min)**	** style="border-bottom: thin solid #000000;" MS/MS transition (dMRM)**		**Fragementor voltage (V)**	**Collision energy (V)**
			**Precursor ion (*m/z*)**	**Product ion (*m/z*) (quantifier/qualifier)**		
GSE capsule	gallic acid	1.28	169	125.0/79.1	86	12/24
	catechin	2.05	289.1	245.2/123.1	120	12/36
	procyanidin B2	2.21	577.1	289.0/407.0	145	25/25
	epicatechin	2.34	289.1	245.2/203.1	134	12/20
RSV capsule	*trans*-resveratrol	1.67	227.1	185.0/143.0	115	17/29

#### RSV Capsule Dissolution Samples (LC-MS Method)

For LC-MS parameters, mobile phase A was 0.1% FA in water, and mobile phase B was 0.1% FA in can with a flow rate of 0.4 ml/min. The gradient was 25 to 60% B in 3 min, and the column was equilibrated with 25% B for 0.5 min between injections. The injection volume was 1 μl. The column was thermostatted at 30°C, and the autosampler was set to 4°C. Nitrogen was used as the nebulizing and drying gas. The nebulizer was set to 35 psi and the drying gas to 300°C with a flow rate of 12 L/min, while the sheath gas was set to 250°C with a flow rate of 12 L/min. The nitrogen used for MS electrospray ionization was generated from a Parker Balston NitroFlow60NA nitrogen generator.

All the RSV dissolution samples were diluted using a solution of 70% methanol acidified using 0.1% formic acid (1:100). The prepared sample was centrifugated at 12,000 rpm for 10 min. The supernatant was directly injected into UHPLC. The scan mode was dMRM. The parameters are presented in Table 1. Brown Eppendorf tubes were used to protect the RSV dissolution samples from light.

## Results

### Calculation of GSE Marker Compounds Dissolved

**Figure 1:**
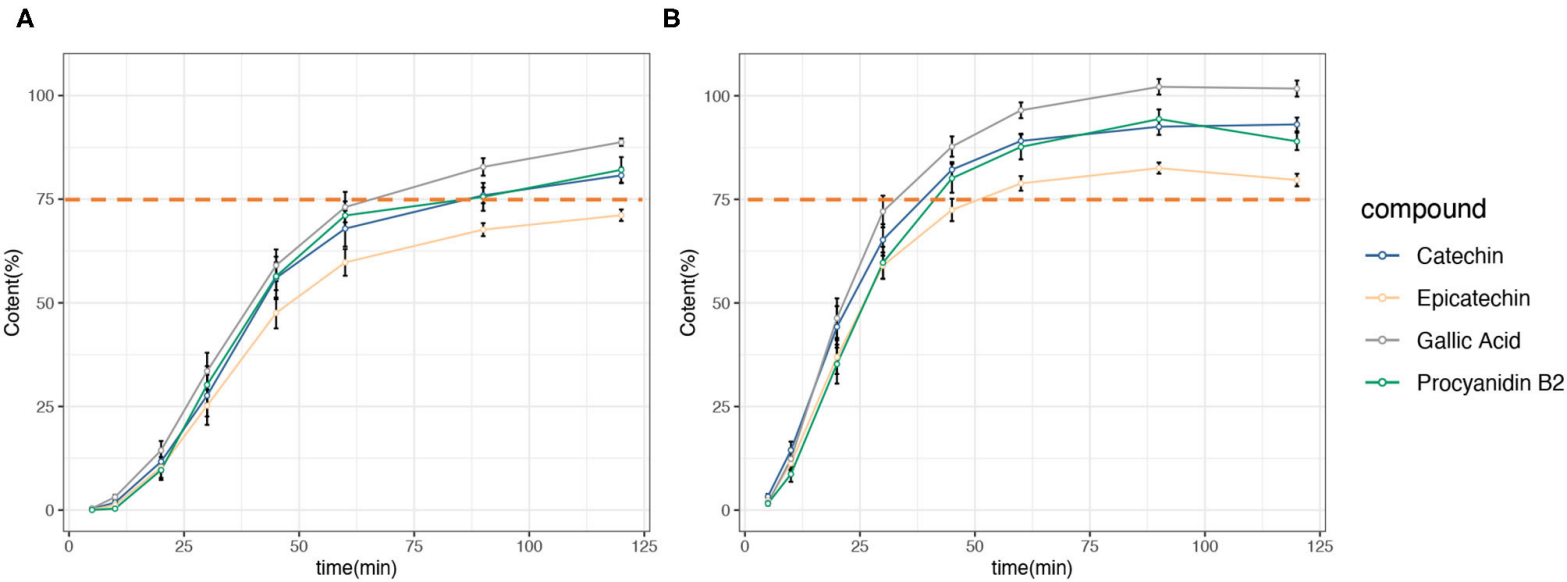
Dissolution profile of grape seed extract (GSE) capsules showing the four major components: catechin, epicatechin, gallic acid, and procyanidin B2. **(A)** 0.05 M acetate buffer (pH 4.6). **(B)** 0.1 N hydrochloric acid (pH 1.2).

For all the tested GSE capsules, the percentage of four marker compounds (gallic acid, catechin, epicatechin, and procyanidin B2) released from GSE capsules was calculated based on analytically measured amounts. The dissolution profile is presented in Figure 1, and the detailed data are shown in Supplementary Material. In general, the amount of four marker compounds in both the dissolution media increased rapidly. For the 0.1N HCl acid medium, the GSE capsules released 96.49, 89.09, 87.65, and 78.84% of gallic acid, catechin, procyanidin B2, and epicatechinin, respectively, within 60 min, meeting the general chapter USP disintegration and dissolution standards. However, for the acetate buffer with a pH of 4.6, the GSE capsules released 73.09, 67.9, 71.06, and 59.75% of gallic acid, catechin, procyanidin B2, and epicatechin, respectively, within 60 min, failing to meet the USP guidelines of 75% release within 60 min.

### Calculation of RSV Marker Compounds Dissolved

**Figure 2:**
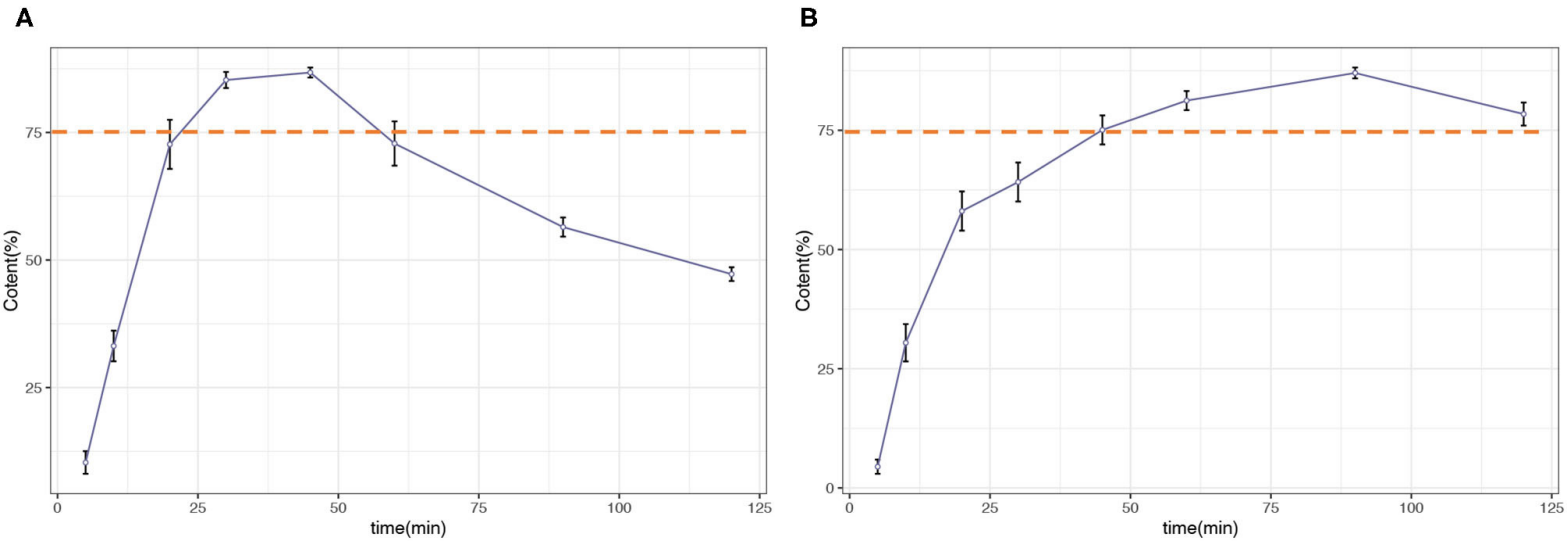
Dissolution profile of resveratrol (RSV) capsules. **(A)** 0.1 N hydrochloric acid (pH 1.2). **(B)** 0.05 M acetate buffer (pH 4.6).

The percentage of trans-RSV released from the RSV capsules was also calculated based on the LC-MS data. The dissolution profile is illustrated in Figure 2, and detailed data are shown in Supplementary Material. For this case, the amount of trans-RSV in both dissolution media increased rapidly, and the release was >75% within 60 min, meeting the general chapter USP disintegration and dissolution standards.

## Discussion

In this study, a novel dissolution test and LC/MS analysis were developed to evaluate the performance of grape polyphenol dietary supplements, namely, GSE and RSV capsules.

**Figure 3:**
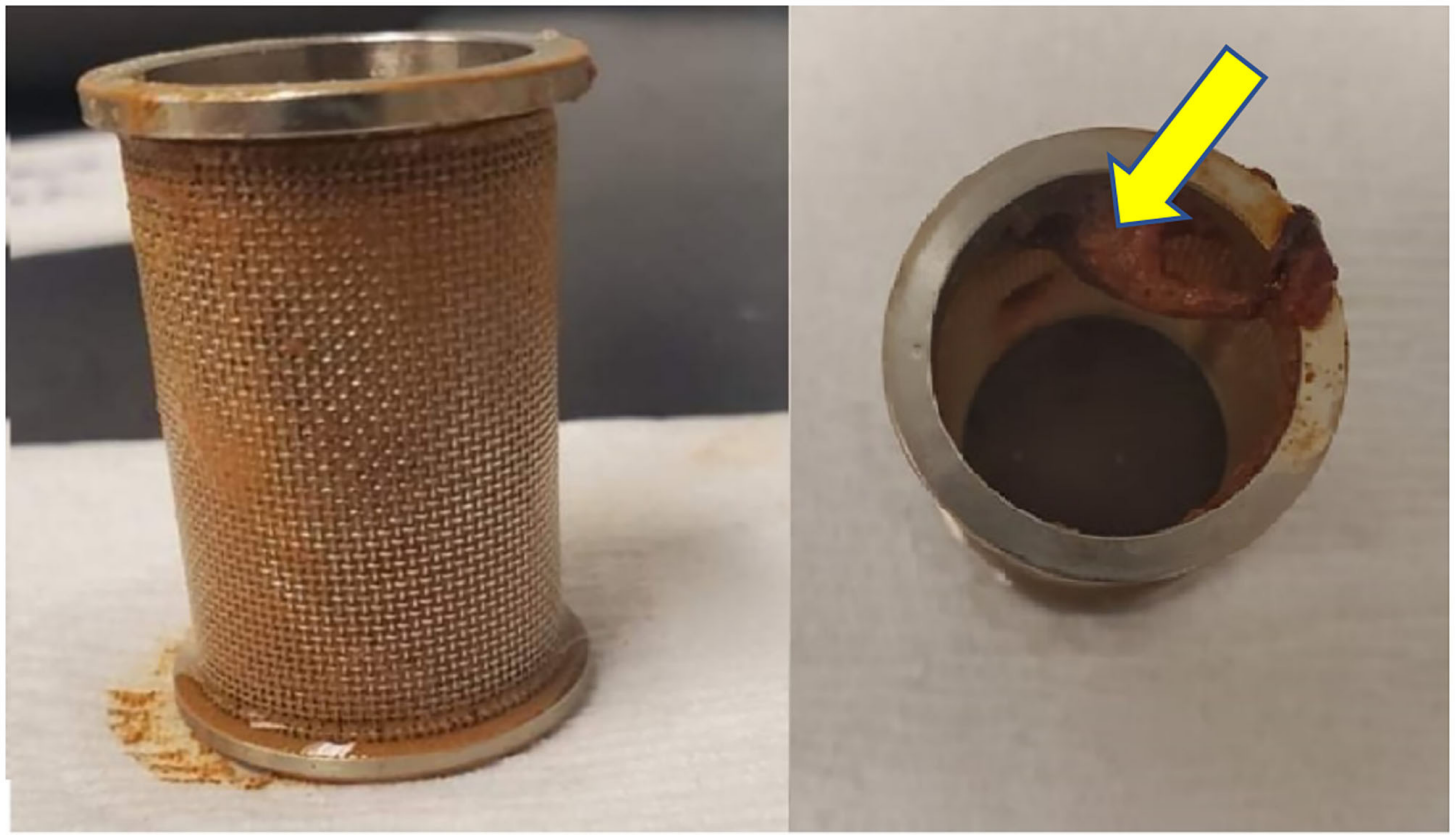
GSE capsule residues within the dissolution basket after 2 h dissolution in 0.1 N hydrochloric acid (pH 1.2) (as indicated by arrowheads).

For the GSE capsules, the marker compounds released rapidly in both media and under conditions found in the stomach (at pH 1.2) met the USP dissolution guidelines. However, the same GSE capsules failed to meet that USP guideline of a 75% release within 60 min in the acidic medium with a pH of 4.6.The reasons for dissolution test failure can be broadly classified into two categories: dissolution procedure and capsule quality (12). It is possible that these marker compounds may be extracted less efficiently with the dissolution media than with sonicated organic solvent mixtures. Also, in this study, dissolution method type I was used where the capsules were placed inside the basket with a 40-mesh size. The openings of the 40-mesh size may be small to allow the release of all dissolved products. Any coacervate, which could result during the disintegration and dissolution of GSE capsules in 0.1 N hydrochloric acid (pH 1.2), cannot pass into the bulk dissolution medium, as shown in Figure 3. If capsule residues could not completely pass through the basket into the dissolution medium, the percentage of dissolved GSE components would be lower than expected and would finally lead to incomplete release (<100%). It is worth noting that using a spiral capsule sinker with large opening (10-mesh size) could solve this problem. Moreover, a previous study has reported that some products were sensitive to chosen test conditions, namely, beaker size and the equipment used in dissolution study (13). Because of the limitation of our laboratory and costs, we did not optimize these parameters, and these aspects might be further improved. The results also indicate that the bioactive compounds may be not released properly from GSE capsules in the 0.05 M acetate buffer (pH 4.6), while these compounds were successfully released from GSE capsules in 0.1 N hydrochloric acid (pH 1.2).

For the RSV capsules, trans-RSV released was more than 75% within 60 min in both the dissolution media. However, for the 0.1-N HCl acid medium, the amount of trans-RSV dropped after 45 min. Due to the stability and solubility of trans-RSV being highly influenced by pH and temperature, trans-RSV might be degraded during the experiment. Hence the concentration obtained by UHPLC-QqQ was much lower than the amount that was released. Photochemical and photocatalytic degradation of trans-RSV is another possible reason for the reduction, owing to cis-isomerization, which occurs when the *trans*-isomer is exposed to sunlight, or artificial or natural UV radiation at a wavelength of 254 or 366 nm (14–18). In this study, even though the dissolution apparatus was covered with tin foil to protect RSV from light, and brown Eppendorf tubes were used when preparing the RSV capsule samples, light-sensitive trans-RSV may still be exposed to visible light during the experiment. Hence, it is worthwhile to note that special attention must be paid to trans-RSV dissolution testing. These results demonstrate the need to improve the dissolution apparatus for these light-sensitive compounds. Our findings suggest that product formulation needs to be considered in all such studies that examine the bioavailability of a botanical product in animal or human trials and in as rigorous a manner as botanical authentication and chemical profiling of the actual product. That is, the material of the capsule itself and the excipients used and blended into the actual botanicals need to be subjected to such dissolution tests to ensure that the correct concentrations needed in animal and human studies are delivered.

## Data Availability Statement

The original contributions presented in the study are included in the article/Supplementary Material, further inquiries can be directed to the corresponding authors.

## Author Contributions

WL, TO, FM, JS, and QW designed the study. WL, TO, and HP conducted the experiments, performed data analysis, and drafted the manuscript. FM, JS, and QW supervised the overarching project, and all the co-authors, namely, DR, MF, GP, and JM, discussed the original concept and overall objectives with DR and MF providing key insights into botanical ingredients and formulation. GP and JM provided criteria under which the botanicals were needed for applications in the planned clinical trials. All authors contributed to the article, reviewed and strengthened the final version of the manuscript, and approved the submitted version.

## Funding

This study was supported by grant U19 AT010835 from the Office of Dietary Supplements (ODS), the National Center for Complementary and Integrative Health (NCCIH), and the National Institute on Aging (NIA) of the NIH in support of *Influence of Dietary Botanical Supplements on Biological and Behavioral Resilience* awarded to the Icahn School of Medicine at Mount Sinai. GP holds a Senior Scientist Award.

## Conflict of Interest

DR was employed by company Eagle Nutritionals. The remaining authors declare that the research was conducted in the absence of any commercial or financial relationships that could be construed as a potential conflict of interest.

## Publisher's Note

All claims expressed in this article are solely those of the authors and do not necessarily represent those of their affiliated organizations, or those of the publisher, the editors and the reviewers. Any product that may be evaluated in this article, or claim that may be made by its manufacturer, is not guaranteed or endorsed by the publisher.
